# Retention of low-fitness genotypes over six decades of admixture between native and introduced tiger salamanders

**DOI:** 10.1186/1471-2148-10-147

**Published:** 2010-05-18

**Authors:** Jarrett R Johnson, Benjamin M Fitzpatrick, H Bradley Shaffer

**Affiliations:** 1 Department of Evolution and Ecology & Center for Population Biology, University of California, Davis, CA 95616, USA; 2 Department of Ecology and Evolutionary Biology, University of Tennessee, Knoxville, TN 37996, USA

## Abstract

**Background:**

Introductions of non-native tiger salamanders into the range of California tiger salamanders have provided a rare opportunity to study the early stages of secondary contact and hybridization. We produced first- and second-generation hybrid salamanders in the lab and measured viability among these early-generation hybrid crosses to determine the strength of the initial barrier to gene exchange. We also created contemporary-generation hybrids in the lab and evaluated the extent to which selection has affected fitness over approximately 20 generations of admixture. Additionally, we examined the inheritance of quantitative phenotypic variation to better understand how evolution has progressed since secondary contact.

**Results:**

We found significant variation in the fitness of hybrids, with non-native backcrosses experiencing the highest survival and F2 hybrids the lowest. Contemporary-generation hybrids had similar survival to that of F2 families, contrary to our expectation that 20 generations of selection in the wild would eliminate unfit genotypes and increase survival. Hybrid survival clearly exhibited effects of epistasis, whereas size and growth showed mostly additive genetic variance, and time to metamorphosis showed substantial dominance.

**Conclusions:**

Based on first- and second- generation cross types, our results suggest that the initial barrier to gene flow between these two species was relatively weak, and subsequent evolution has been generally slow. The persistence of low-viability recombinant hybrid genotypes in some contemporary populations illustrates that while hybridization can provide a potent source of genetic variation upon which natural selection can act, the sorting of fit from unfit gene combinations might be inefficient in highly admixed populations. Spatio-temporal fluctuation in selection or complex genetics has perhaps stalled adaptive evolution in this system despite selection for admixed genotypes within generations.

## Background

Whether natural or anthropogenic in origin, zones of secondary contact provide a powerful test of the compatibility of previously allopatric species, and lend insight into the mechanisms responsible for reproductive isolation and speciation. When reproductive isolation is incomplete and secondary contact results in the formation of a hybrid zone, the resulting dynamics also give us a glimpse into the genetics underlying phenotypic divergence and the evolution of reproductive isolating mechanisms. On those rare occasions when we observe the early generations of contact, we can also examine ecological and genetic changes as they unfold, providing a view of short-term dynamics that is not available in most well-established, natural hybrid zones.

Hybridization between two species has many potential evolutionary consequences [e.g., [1]]. At one extreme, hybridization may end in the fusion of two lineages in which speciation has failed to occur fully [2,3]. In this case, the homogenizing process of gene flow may erase the genetic signature of earlier lineage diversification and subsequent reticulate evolution. Alternatively, two lineages in secondary contact may create a stable hybrid zone in which parental taxa maintain separate evolutionary trajectories outside of the hybrid zone but continue to produce dysfunctional hybrids in the contact zone [4,5]. Finally, hybridization can lead to increased genetic variation and evolutionary novelty in the form of recombinant genotypes [6-8]. In this situation, even when average fitness is quite low, hybrid populations may produce a few highly successful recombinant genotypes across all or a subset of ecological backgrounds, which can breed true and increase in frequency. When hybridization occurs as the result of introductions of an exotic species, the evolutionary outcomes are somewhat modified because ongoing gene flow from the introduction source is limited or absent [9,10]. Homogenization of genotypes can still occur with the introgression of introduced alleles and loss of native variants, but the effects on parental populations are asymmetrical since gene flow from "pure" individuals is unidirectional (assuming that there are not frequent subsequent introductions of non-native individuals).

In either natural or human-mediated hybrid zones, exceptionally fit individuals have the potential to establish new evolutionary lineages, either displacing one or both parental lineages or diverging into a new ecological zone [7,11-13]. The realization of this potential depends on the strength of selection, the number of genes involved in fitness variation, and the mode of action of those genes (additive, dominance, or epistasis). For example, a strong initial barrier to gene flow in the first generation (F1) of secondary contact will slow the establishment and spread of fit recombinant genotypes that may arise in subsequent generations, whereas F1 heterosis may speed up this process. Further, if the beneficial fitness effects of an allele in one genetic background do not outweigh deleterious effects or correlated selection in another genetic background, polymorphism may be eliminated before fit recombinant genotypes can reach a high enough frequency to be maintained by selection. Thus, the fitness consequences of the initial stages of hybridization, which are virtually always unknown in natural hybrid zones, can have profound effects on the longer-term consequences of hybridization.

The mode of gene action underlying particular phenotypes also plays an important role in the potential of hybrid populations to maintain genetic diversity and respond to selection [14]. Phenotypes with largely additive inheritance express main effects across different genetic backgrounds whereas traits experiencing strong epistatic effects vary depending on the interaction of other genetic factors [15-17], and traits affected largely by dominance typically exhibit striking phenotypic differences within cross types [18,19]. Therefore, traits with high dominance or epistatic variance might not respond to selection, even when they have large fitness effects, and the probability and rate of adaptive evolution in hybrid populations might be constrained by the genetic basis of transgressive traits.

For decades, secondary contact between species mediated by incidental human transport or deliberate introduction has been recognized as potentially detrimental to native species [20-23]. When introductions result in hybridization with native species, new dimensions are added to conservation issues. While hybridization and subsequent introgression has been characterized as a threat simply because replacement of native by introduced alleles is philosophically undesirable [12,24], or might compromise the legal status of protected native species [25], more objectively detrimental impacts are also possible. Hybridization can create novel invasive phenotypes with negative ecological impacts [26-28], or hybrid dysfunction might make admixed populations more vulnerable to extinction [12,29,30]. For these reasons, studies of the evolutionary and ecological changes set in motion by hybridization have both applied and theoretical significance.

One of the key issues facing empirical analyses of hybrid zones is that the initial evolutionary dynamics of secondary contact are often obscured by many generations of admixture. Our understanding of hybrid zones is dominated by examples of secondary contact that have not resulted in extinction of one or both parental species or reinforcement of reproductive isolation, since these are the situations most often identified in the wild. Analyses of these zones have provided tremendous insights into the genes and characters that remain differentiated in the face of hybridization, but they are potentially a biased subset of the genes and characters that differed *prior *to secondary contact [31].

Our research on a recently established, human-mediated hybrid zone offers the rare opportunity to observe the initial dynamics of secondary contact between gene pools formerly separated ~5 mya [32]. In the 1940s, bait dealers introduced thousands of the barred tiger salamander (BTS; *Ambystoma tigrinum mavortium*) from the Great Plains of the US into the range of the native California tiger salamander (CTS; *A. californiense*)[33]. Riley et al. [33] report (based on discussions with some of the individuals responsible for the introductions) that California's emerging bass (*Micropterus *spp.) fishing industry and known life history variation between the salamander species motivated the introductions. Bass fishermen use larval tiger salamanders (waterdogs) for bait, and they favor large larvae to catch large bass. CTS metamorphose at small sizes and have a relatively short larval period during which time they can be harvested from wild ponds. In contrast, BTS have a highly plastic larval period and can remain aquatic and attain very large sizes if ponds are permanent, providing a source of bait that is both larger and potentially available year-round. These intentional introductions have resulted in a large number of hybrid populations within the Salinas Valley of California. While there have been many generations of admixture in the heart of the Salinas Valley hybrid swarm, there has not been enough time for any nonnative allele to become fixed throughout the range of the native species [34]. Furthermore, the success of hybrid genotypes appears heavily influenced by local environmental conditions, with anthropogenic changes in breeding habitat supporting increasingly non-native admixed populations [35,36].

We have two main objectives in this study. First, we provide a direct comparison of viability among first- and second-generation hybrids to determine the strength of the initial barrier to gene exchange. Given that we have observed both hybrid dysfunction and vigor in contemporary hybrid populations [37,38], it is not clear what the fitness of F1 hybrids may have been when they first appeared 50-60 years ago. Second, we examine the extent to which natural selection has affected mean fitness in a contemporary hybrid population. If hybridization is always an important source of variation for adaptation [39-42], we expect to see the mean fitness of admixed populations increase following the initial mixture, particularly if the early hybrid generations show hybrid dysfunction.

To investigate whether there was a significant barrier to gene flow during the initial stages of hybridization, we performed breeding crosses and individually reared in the laboratory all possible combinations of first- and second-generation hybrids that could have resulted from the initial contact of CTS and BTS. Simultaneously, we examined contemporary-generation individuals that were collected from the wild as larvae, bred in the laboratory, and reared to maturity. This experimental approach allows us to describe the genetics of phenotypic variation, understand the intrinsic effects of recombination resulting from hybridization, and evaluate the result of approximately 20 generations of genomic admixture in the wild.

## Results

We reared 14 first- and second-generation line-cross families and 5 contemporary-generation (~20^th ^generation) families of salamanders from embryos to metamorphosis under standard laboratory conditions. The early-generation families were comprised of pure native CTS (N = 2 replicate families), pure introduced BTS (N = 2), F1 (N = 2), backcrosses to CTS (bcCTS; N = 3), backcrosses to BTS (bcBTS; N = 3), and F2 (N = 2) line crosses. We measured survival to metamorphosis and fitness-related traits (mass, snout-vent length [SVL], time-to-metamorphosis [Tmet], and growth) at metamorphosis. Means and variances for each line cross are presented in Table 1.

**Table 1:** Character means () and variances (*s*^*2*^) for each line cross.

			Mass		SVL		Tmet		Growth		Survival	
			
Cross	*N*_*fam*_	*N*_*ind*_		*s*^*2*^		*s*^*2*^		*s*^*2*^		*s*^*2*^		*s*^*2*^
CTS	2	33	2.222	0.020945	4.161	0.002270	21.402	0.172576	1.306	0.001135	0.694	0.057741
BTS	2	40	3.041	0.113293	4.427	0.009838	22.397	1.229224	1.483	0.004610	0.918	0.000006
bcCTS	3	61	2.374	0.014341	4.200	0.003334	21.401	0.396139	1.336	0.000225	0.753	0.027811
bcBTS	3	77	2.953	0.012654	4.392	0.003761	21.905	0.371182	1.467	0.000295	0.953	0.004133
F1	2	89	2.615	0.001193	4.279	0.000174	20.876	0.019523	1.399	0.000032	0.849	0.033349
F2	2	37	2.474	0.043597	4.233	0.004615	21.513	0.122002	1.358	0.001693	0.617	0.000007
Contemporary Hybrids	5	77	2.627	0.035355	4.291	0.003233	22.135	0.680014	1.382	0.000876	0.550	0.035057

Mass and snout-vent length (SVL) values have been log transformed (ln [*x *+ 1]). Time to metamorphosis (Tmet) has been square root transformed. Growth data were obtained by dividing 'mass' by 'Tmet'. Survival data represent the proportion of individuals reaching metamorphosis.

### Quantitative Genetics

**Figure 1:**
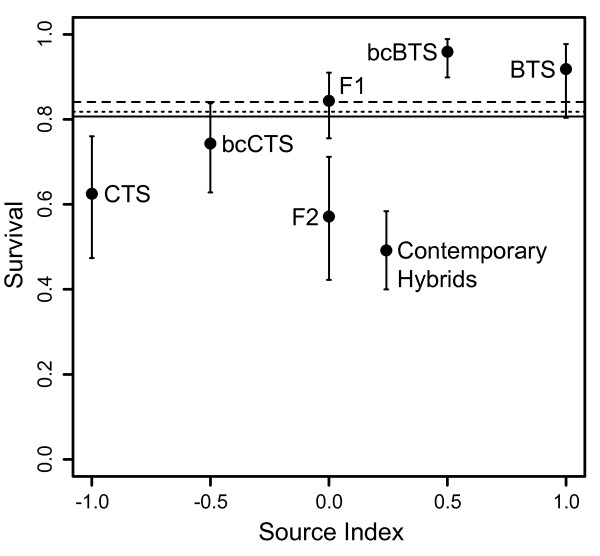
**Comparison of line - cross means to weighted expected mean survival in the 0th (solid line), 1st (dashed line), and 2nd (dotted line) generations of admixture (± 95% CI)**. The 'Source Index' (*θ*S) = 2P - 1, where P is the average frequency of introduced BTS alleles in each cross type (Lynch 1991).

**Figure 2:**
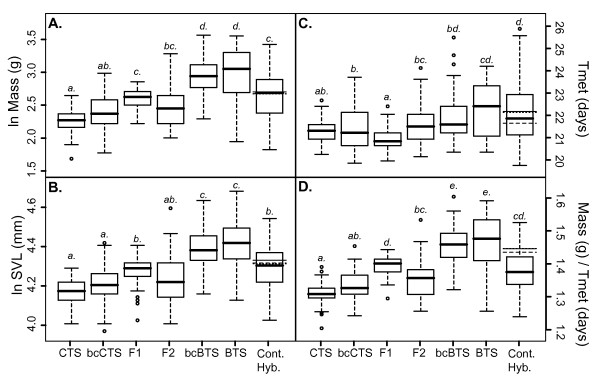
**Box-and-whisker plots of phenotypic trait values for A) Mass, B) SVL, C) Tmet, & D) Growth**. Mass and SVL have been log-transformed and Tmet and Growth have been square-root-transformed. Letters above each line cross denote significant differences at *α *= 0.05 based on multiple t-test comparisons with Bonferroni correction. Horizontal lines denote the expected mean phenotypes for contemporary hybrids in the 0th (solid line segment), 1st (dashed line segment), and 2nd (dotted line segment) generations of admixture. The abbreviation 'Cont. Hyb.' stands for 'Contemporary Hybrids'.

Observed trait means for each parental cross were remarkably different both for survival (Figure 1, Table 1) and secondary fitness-related traits (Figure 2, Table 1) and provided ample among-species divergence within which to compare the phenotypes of hybrid cross types using weighted least squares regression to parameterize quantitative genetic models of gene action. Additional files 1, 2, 3 and 4 depict the fit of observed trait means (± SE) for each line-cross to the multiple regression (solid line) equation for each phenotype, and Additional file 5 shows representatives from each line-cross category. We found very good fits to the additive model for morphological traits such as mass (Table 2, Additional file 1) and SVL (Table 2, Additional file 2). We calculated growth as the mass at metamorphosis divided by the number of days each individual required to reach metamorphosis, and the additive model also best describes the genetic basis of growth differences (Table 2, Additional file 3). Tmet appears to have a strong dominance component to the variation observed (Table 2, Additional file 4). Both linear and binomial regression results agreed regarding epistatic effects on survival (Table 3), a pattern largely driven by low survival of F2 larvae (Figure 1, Table 1).

**Table 2:** Evaluation of the Chi-square test statistic (*χ*^2^) for fit of observed data to expectations under alternative quantitative genetic models.

Trait	Model	*χ*^2^		*P*_Λ_
Mass	*μ*_0_	28.384	0.0080	NA
	*μ*_0 _+ b_S_*	5.979	0.9171	0.0000
	*μ*_0 _+ b_S _+ b_H_	5.917	0.8788	0.8038
	*μ*_0 _+ b_S _+ b_H _+ b_SS_	5.533	0.8528	0.5357
	*μ*_0 _+ b_S _+ b_H _+ b_SS _+ b_HH_	4.791	0.8522	0.3888
	*μ*_0 _+ b_S _+ b_H _+ b_SS _+ b_HH _+ b_SH_	4.157	0.8427	0.4262

SVL	*μ*_0_	8.714	0.7942	NA
	*μ*_0 _+ b_S_*	2.163	0.9991	0.0105
	*μ*_0 _+ b_S _+ b_H_	2.129	0.9980	0.8530
	*μ*_0 _+ b_S _+ b_H _+ b_SS_	2.042	0.9960	0.7688
	*μ*_0 _+ b_S _+ b_H _+ b_SS _+ b_HH_	1.795	0.9943	0.6193
	*μ*_0 _+ b_S _+ b_H _+ b_SS _+ b_HH _+ b_SH_	1.527	0.9923	0.6042

Tmet	*μ*_0_	45.252	0.0000	NA
	*μ*_0 _+ b_S_	36.341	0.0003	0.0028
	*μ*_0 _+ b_S _+ b_H_*	17.657	0.0899	0.0000
	*μ*_0 _+ b_S _+ b_H _+ b_SS_	17.163	0.0708	0.4821
	*μ*_0 _+ b_S _+ b_H _+ b_SS _+ b_HH_	16.781	0.0523	0.5368
	*μ*_0 _+ b_S _+ b_H _+ b_SS _+ b_HH _+ b_SH_	16.782	0.0325	1.0000

Growth	*μ*_0_	6.568	0.9230	NA
	*μ*_0 _+ b_S_*	1.316	0.9999	0.0219
	*μ*_0 _+ b_S _+ b_H_	1.317	0.9998	1.0000
	*μ*_0 _+ b_S _+ b_H _+ b_SS_	1.137	0.9997	0.6720
	*μ*_0 _+ b_S _+ b_H _+ b_SS _+ b_HH_	0.968	0.9995	0.6811
	*μ*_0 _+ b_S _+ b_H _+ b_SS _+ b_HH _+ b_SH_	0.757	0.9994	0.6457

The first model (including only *μ*_0_) is a null model of no variation among cross types. Significant chi-square values ( < 0.05) indicate lack of fit relative to a saturated model. Significant (*P*_Λ _< 0.05) likelihood-ratio tests (Eq. 2) indicate improvement in fit of higher-order models over simpler models. Asterisks denote the best-fit model for each phenotype.

**Table 3:** Model comparison for linear mixed-effect logistic regression on alternative quantitative genetic models for survival of cross types.

Model	df	AIC	BIC	Λ	*χ*^2^		
*μ*_0 _+ b_S_	2	362.89	370.89	-179.44			
*μ*_0 _+ b_S _+ b_H_	3	364.69	376.70	-179.34	0.1978	1	0.6565
*μ*_0 _+ b_S _+ b_H _+ b_SS_	4	349.63	365.64	-170.81	17.0631	1	0.0000
*μ*_0 _+ b_S _+ b_H _+ b_SS _+ b_HH_*	5	345.30	365.32	-167.65	6.3258	1	0.0119
*μ*_0 _+ b_S _+ b_H _+ b_SS _+ b_HH _+ b_SH_	6	345.49	369.51	-166.74	1.8142	1	0.1780

Models are evaluated using Akaike's Information Criterion (AIC), Bayesian Information Criterion (BIC), and Log Likelihood (Λ) goodness-of-fit estimates. The best model is denoted by an asterisk. Chi-square and p-values compare the respective model to the simpler model in the row above.

### Initial barrier to gene flow

Our F1 generation represents a reenactment of the initial postzygotic barriers to reproduction between the native and introduced salamanders when BTS were first introduced. Multiple comparison t-tests (Table 4) demonstrate that F1 phenotypes are intermediate to and significantly different from the parental crosses for all morphological traits measured (mass, SVL, and growth), which is consistent with the inference of an additive genetic basis for divergence of these traits. For Tmet, which shows a significant dominance effect, F1 crosses are not significantly different from native CTS but reach metamorphosis significantly faster than introduced BTS (Table 4).

**Table 4:** Table of *P*-values for multiple comparisons with t-tests (below diagonal), and pair-wise mean differences (column header minus row name; above diagonal).

A. Mass	CTS	BTS	F1	F2	bcCTS	bcBTS	PondH
CTS	--	12.8	4.4	3.0	1.7	10.4	5.4
BTS	<0.0001*	--	-8.4	-9.8	-11.1	-2.4	-7.4
F1	<0.0001*	<0.0001*	--	-1.4	-2.7	6.0	1.0
F2	0.0002*	<0.0001*	0.0264	--	-1.3	7.4	2.4
bcCTS	0.0236	<0.0001*	<0.0001*	0.0562	--	8.7	3.7
bcBTS	<0.0001*	0.3273	<0.0001*	<0.0001*	<0.0001*	--	-5.0
PondH	<0.0001*	<0.0001*	0.6876	0.0131	<0.0001*	<0.0001*	--
**B. SVL**	**CTS**	**BTS**	**F1**	**F2**	**bcCTS**	**bcBTS**	**PondH**
CTS	--	19.9	8.0	5.1	2.7	16.8	9.2
BTS	<0.0001*	--	-11.9	-14.7	-17.2	-3.0	-10.7
F1	<0.0001*	<0.0001*	--	-2.8	-5.3	8.9	1.2
F2	0.0033	<0.0001*	0.0400	--	-2.4	11.7	4.1
bcCTS	0.0927	<0.0001*	<0.0001*	0.1007	--	14.1	6.5
bcBTS	<0.0001*	0.2361	<0.0001*	<0.0001*	<0.0001*	--	-7.6
PondH	<0.0001*	<0.0001*	0.3750	0.0071	<0.0001*	<0.0001*	--

**C. Tmet**	**CTS**	**BTS**	**F1**	**F2**	**bcCTS**	**bcBTS**	**PondH**
CTS	--	11	-6	1	0	6	8
BTS	0.0002*	--	-17	-10	-11	-6	-3
F1	0.0314	<0.0001*	--	7	6	11	14
F2	0.3585	0.0031	0.0008*	--	-1	4	7
bcCTS	0.5792	0.0002*	0.0008*	0.6311	--	6	8
bcBTS	0.0068	0.0883	<0.0001*	0.0842	0.0095	--	3
PondH	0.0001*	0.7380	<0.0001*	0.0023*	<0.0001*	0.0974	--

**D. Growth**	**CTS**	**BTS**	**F1**	**F2**	**bcCTS**	**bcBTS**	**PondH**
CTS	--	0.09	0.04	0.02	0.01	0.08	0.04
BTS	<0.0001*	--	-0.05	-0.07	-0.08	-0.01	-0.06
F1	<0.0001*	<0.0001*	--	-0.02	-0.03	0.04	-0.01
F2	0.0001*	<0.0001*	0.0003*	--	-0.01	0.06	0.01
bcCTS	0.0290	<0.0001*	<0.0001*	0.0291	--	0.07	0.02
bcBTS	<0.0001*	0.3504	<0.0001*	<0.0001*	<0.0001*	--	-0.04
PondH	<0.0001*	<0.0001*	0.0310	0.0587	<0.0001*	<0.0001*	--

Asterisks denote significance (a = 0.05) following Bonferroni correction.

Most important in terms of overall fitness, F1 crosses had intermediate survival, showing no evidence of hybrid dysfunction (Table 5), although they do show relatively high embryonic (*i.e*., pre-hatching) mortality [34]. Thus, the current results indicate that larval mortality (*i.e*., post-hatching through completion of metamorphosis) probably did not contribute any additional barrier to gene flow during the onset of secondary contact between CTS and BTS (Figure 1). Larval mortality was greater in the F2 families than in the F1, introduced BTS, and bcBTS, but was not significantly different from native CTS and bcCTS (Table 5, Figure 1). Of the six classes of possible first and second-generation crosses, bcBTS had the greatest larval survival (Figure 1).

**Table 5:** Fisher's exact tests for multiple comparisons with sequential Bonferroni correction (below diagonal) and mean weighted percent survival differences between cross types (column header - row names; above diagonal).

	CTS	BTS	F1	F2	bcCTS	bcBTS	PondH
CTS	--	0.330	0.282	-0.051	0.155	0.425	-0.073
BTS	0.0006*	--	-0.049	-0.382	-0.176	0.095	-0.404
F1	0.0057	0.2990	--	-0.333	-0.127	0.143	-0.355
F2	0.6801	0.0001*	0.0005*	--	0.206	0.477	-0.022
bcCTS	0.2262	0.0178	0.1234	0.0520	--	0.270	-0.228
bcBTS	<0.0001*	0.4413	0.0077	<0.0001*	<0.0001*	--	-0.499
PondH	0.1278	<0.0001*	<0.0001*	0.3990	0.0006*	<0.0001*	--

Asterisks denote significance based on sequential Bonferroni correction critical values.

### Contemporary hybrid fitness

Our contemporary hybrid crosses represent the result of selection in the wild after ~20 generations of interbreeding and consequent natural selection. Multiple comparisons reveal that these contemporary hybrids have not significantly diverged from F2 crosses with respect to morphological traits (mass, SVL, and growth), but are significantly different from each backcross (Table 4, Figure 2). Again, this pattern is consistent with our interpretation that morphological traits have largely additive differences. However, contemporary hybrids are significantly slower to metamorphosis (larger Tmet value) than either F2 crosses or backcrosses to CTS, and appear more similar to backcrosses to BTS in this trait (Table 4). Tmet is an important life history characteristic that can have major effects on fitness in landscapes with variable pond hydroperiods, and selection at our contemporary population appears to be favoring an increase in Tmet relative to F2 crosses. However, Tmet and size-related means for contemporary hybrids did not differ substantially from the predicted values based on the first two generations of admixture (Figure 2). Interestingly, mean growth for contemporary hybrids was lower than expected (Figure 2D) indicating that extended time-to-metamorphosis is not resulting in increased size-at-metamorphosis. Similarly, contemporary hybrids experienced the lowest mean survival (similar to that of F2 families; Table 5), and significantly lower than the expected weighted averages predicted based on early admixture (Figure 1). That is, mean survival of contemporary hybrid larvae is lower than mean survival in the first two generations of interbreeding, assuming equal admixture proportions.

## Discussion

### Quantitative genetics

Phenotypic differences between each parental species and their hybrids are both statistically and biologically significant, and appear to be affected by a combination of additive, dominance and epistatic gene action. Specifically, we observed strong additive effects for each trait measured, and a simple additive model was sufficient to explain divergence in important body size attributes (*e.g*., snout-vent length, mass, growth). However, differences among cross types in Tmet required the inclusion of dominance effects to adequately explain the variation we observed. Variation in survival among the cross types was best explained when epistatic interactions were included in the analyses. Hybrid breakdown was evident in the F2 larvae, but the effect was weak and not likely to present a major barrier to admixture. More interesting is the persistence of low viability in the contemporary hybrid families. This observation runs counter to our expectation that natural selection would effectively remove low-fitness gene combinations from hybrid populations and implies the existence of important genetic or ecological constraints on the ability of natural selection to distill true-breeding, high-fitness recombinant genotypes from hypervariable admixed populations.

Many previous studies have documented phenotypic differences arising from multiple genes acting additively on phenotypic traits [43-45], and our analyses add another example to this body of work. Our study also contributes to the growing body of literature that describes the importance of non-additive gene action for evolutionary processes such as adaptation and speciation [19,43,46]. Our results suggest that higher-order gene interactions may also be important in determining the outcome of hybridization between species in secondary contact, because some of the most important determinants of salamander fitness (*i.e*., survival and Tmet) demonstrated significant non-additive inheritance.

### Initial barrier to gene flow

To understand the strength of the initial barrier to gene flow we compared F1 hybrid fitness to pure parental crosses. Previous work in this system has documented larval hybrid vigor both in field studies of admixed contemporary populations [38] and experimental mesocosms [27], but has also demonstrated high embryonic mortality [34]. We assessed the fitness of F1 individuals in several ways. With respect to the most important measure of fitness (survival), the F1 trait mean was intermediate to the parental lines with native CTS salamanders experiencing the greatest risk of mortality and introduced BTS experiencing the lowest. These data may appear at odds with the finding of Fitzpatrick and Shaffer [38] that viability selection in the wild favors the most genetically intermediate individuals, regardless of whether the pre-selection population is highly native or nonnative. However, Fitzpatrick and Shaffer [38] examined contemporary hybrids after ~20 generations of admixture and selection. In these wild populations, completely heterozygous F1 individuals almost certainly no longer exist. Thus, what Fitzpatrick and Shaffer [38] demonstrated is that selection on a given larval cohort seems to favor admixed individuals at the expense of highly native and highly nonnative contemporary hybrids. Similarly in our lab crosses, we see increased larval survival for our backcrosses relative to their respective parental lines (*i.e*., bcCTS compared to CTS, and bcBTS compared to BTS), which supports the inference that hybrid vigor during the first few generations facilitated the establishment of this hybrid swarm.

The low survival of native CTS salamanders was surprising, as we expected that the parental crosses (which are essentially controls) would both experience high survival. There are two interpretations that might explain the observed mortality of CTS: (1) Our observed mortality is within the range typically experienced in natural CTS populations; or (2) Our husbandry practices are inappropriate for CTS. Survival estimates have not been reported in the literature for wild CTS larvae in non-admixed populations, and even if they had been, competition and predation would make the survival estimates difficult to compare to our lab data. With respect to the latter option, our husbandry techniques have been in use for decades for the breeding and rearing of related ambystomatid salamanders [47]. While it is certainly conceivable that CTS requirements differ in some way from the other tiger salamander species that have been reared using standard protocols, we currently have no means of evaluating that possibility. In either case, our results support a general pattern of CTS experiencing lower survival than hybrids in artificial pond mesocosm experiments [27], field enclosures in wild ponds [48], and admixed wild populations [38] that convince us that the laboratory data, while surprising in the magnitude of mortality experienced by CTS, conforms with our expectations regarding performance relative to hybrid types.

Other fitness-related traits (*i.e*., mass, SVL, growth, and Tmet) demonstrate an interesting pattern with respect to the F1 lines. Morphologically, F1 individuals were intermediate between the parental lines at metamorphosis, yet they metamorphosed earlier than either parental line. When evaluating the effects of our secondary fitness correlates it is important to consider the environmental context of a metamorphosing salamander in California. For example, a faster growth rate with early obligate metamorphosis might be extremely advantageous in short-hydroperiod aquatic habitats, particularly in California's Mediterranean climate where there is practically no possibility of summer rainfall to rescue a drying pond. However, in more permanent aquatic habitats, such as the man-made livestock ponds that are now common in California, delaying metamorphosis would allow the attainment of larger sizes and may be an advantageous strategy [49,50]. Furthermore, avoiding the semi-arid, and often highly agricultural terrestrial habitat that has typified the hybrid swarm region since the initial BTS introduction 60 years ago may further increase fitness of paedomorphic (*i.e*., permanently aquatic) salamanders. Life-history factors have been hypothesized to promote the invasion success of BTS (and BTS-like hybrids) in perennial ponds, where they seem to have a distinct advantage in the field [35]. In the laboratory and the field [27], F1 individuals appear unable to take full advantage of long-hydroperiod aquatic resources by extending the larval growth period, suggesting that fitness gains associated with artificial, perennial breeding sites were not important in the initial success of F1 hybrids. Obligate early metamorphosis might put F1 salamanders at a selective disadvantage in human-modified perennial ponds, but would confer a selective advantage in natural seasonal vernal pools with short hydroperiods.

Survival in second-generation crosses varied dramatically, with bcBTS growing larger and experiencing greater survival than bcCTS or F2 larvae (Figure 1, Table 1). The difference in survival between backcross types (bcCTS vs. bcBTS) is a potentially important distinction when considering the dynamics of hybrid establishment. If the initial non-native introductions were relatively small in magnitude, most F1 hybrids would mate with native CTS and produce bcCTS offspring during the second generation of admixture. Overall, bcCTS animals are quite similar to native CTS with respect to our measurements of fitness (Table 1) and introgression would likely proceed relatively slowly. However, if BTS were repeatedly introduced in large numbers over multiple years (as we have been told was the case by people familiar with the initial introductions in the 1950s), then pure non-native salamanders would have been present in sufficient frequency to result in the production of high-fitness bcBTS families during the second-generation of admixture. The production of bcBTS hybrids likely facilitated the rapid establishment of highly introgressed populations, particularly in the large perennial ponds where the introductions often occurred. However, bcBTS-like families of individuals are unlikely to be produced under scenarios of low-level natural dispersal from the hybrid zone into native CTS populations, resulting in the relatively slow advance of the hybrid swarm that we have observed in nature [36].

### Contemporary hybrid fitness

Given the fitness variation we observed in our first and second generation crosses, the opportunity exists for natural selection to eliminate unfit gene combinations from hybrid populations in the wild, resulting in an increase in mean fitness of contemporary hybrid populations. However, we found that survival for our contemporary hybrid families was significantly lower than introduced BTS, both backcrosses and the F1 crosses. Contemporary hybrid survival was not statistically different from F2 crosses indicating that selection has been unable to remove unfit genotypes from the population over the last 20 generations.

Field measurements indicate that the source of our contemporary hybrid parents (Pond H) has an intermediate mean hybrid index score (HIS; defined as the proportion of nonnative alleles comprising the average genome) of 0.56-0.62 [34,36], corresponding to a source index (*θ*_*S*_) of 0.12-0.24. This is similar to the average values for F2 crosses (HIS = 0.5; *θ*_*S *_= 0), despite 20 generations of recombination and natural selection. There are several possible mechanisms for the persistence of low fitness genotypes in contemporary hybrids despite the apparent fitness benefits experienced by mostly nonnative individuals. First, immigrants from nearby populations with different frequencies of native and non-native alleles could balance the effects of selection [4,6], essentially recreating early-generation admixture phenotypes with reduced survival. Second, fluctuating selection (perhaps based on variation in pond hydroperiod) combined with overlapping generations [51], which is common in CTS populations [52], could create a mixture of sympatric breeding adults that experienced alternative selection regimes as larvae, generating a stable fitness minimum [53,54]. Third, if fitness variation depends largely on dominance and epistatic effects, low-fitness genotypes might be regenerated and high-fitness genotypes broken up by segregation and recombination every generation [17]. A preponderance of non-additive variance can make selection very inefficient at changing allele frequencies [55]. This hypothesis predicts that, for purely genetic reasons, hybridization is unlikely to result in a true-breeding recombinant lineage becoming fixed by positive selection. The potential constraint created by high levels of non-additive variance might apply mostly to highly admixed populations where the inflated level of multilocus genetic variation makes advantageous genotypes especially prone to disruption by recombination. Lower levels of gene exchange might be more efficient at introducing adaptive genotypes into populations.

Of these three explanations for maintenance of low-viability genotypes, the first (continual immigration into a "hybrid sink") is least likely. Although CTS populations readily exchange individuals [56,57], previous genetic surveys of other populations near the Pond H contemporary hybrid population [33,35,36,38] suggest that there are few if any pure CTS populations remaining that could balance the effect of viability selection for increasing introduced allele frequency. On the other hand, these genetic surveys demonstrate that highly nonnative individuals exist in close proximity to our contemporary hybrid site. Given the dramatic increase in survival experienced by the BTS backcrosses, a population receiving frequent highly non-native immigrants should rapidly move towards an equilibrium in which most individuals have highly non-native genomes. Thus, either variation in selection regimes or genetic constraints on the response to selection are the most likely explanations for the retention of variation at molecular markers and the persistence (or recurrence) of hybrid genotypes with low viability. Future comparisons of additional crosses, combined with common-garden-type rearing conditions would help to distinguish between these two hypotheses.

A fourth possibility is that survival in the lab bears little relationship to fitness in the wild. However, our results for contemporary hybrids are consistent with our studies from the wild in that the strong selection on larvae described by Fitzpatrick and Shaffer [38] is possible only if substantial genetic variation in fitness is expressed in contemporary wild populations. Thus, our observations in the wild and the low average viability of contemporary hybrids documented here both strongly conflict with the prediction that ~20 generations of selection in the wild should have eliminated low-fitness genotypes and distilled a genetically stable, high-fitness hybrid lineage.

Localized environment-dependent admixture dynamics have previously been reported in this hybrid swarm [35], with perennial ponds supporting more introduced (*i.e*., higher HIS or *θ*_*S*_) populations. We have also reared a small number (N = 50) of other contemporary hybrids from a perennial site (Johnson Canyon Landfill; JCL) with a HIS of 0.81 near the Pond H contemporary hybrid site. These JCL contemporary hybrid larvae experienced initial mortality (4%) that was similar to pure BTS (8%) and BTS backcross lines (2%) in dramatic contrast to our mortality estimate for lab-reared contemporary hybrid larvae from Pond H (45%). Unfortunately, we cannot include larvae from the JCL site in our analyses because they were not reared to the completion of metamorphosis. Variation in hydroperiod has previously been identified as a major selective force in the evolution of salamanders with complex life history pathways [49,50,58,59]. Our data suggest that in this hybrid swarm, environmental variation (*e.g*., pond hydroperiod) is likely important in balancing selection for highly non-native individuals, and thereby maintaining higher frequencies of native genotypes.

Examples of hybrid speciation and discussion of the potentially creative role of hybridization in evolution [60,61] have been popular in the recent literature. But the generation of highly adaptive recombinant genotypes is likely rare and their success is dependent on a complex combination of extrinsic factors, including the availability of niches unoccupied by either parental species. Therefore the relative importance of hybridization in speciation remains unknown. Our results provide an exciting glimpse at the early dynamics and initial responses to selection of a recently established hybrid system and demonstrate that complex ecology (spatio-temporal fluctuation in selection) or complex genetics (non-additive variance in hybrid fitness) can inhibit or stall adaptive evolution, even when natural selection within generations appears to favor admixed genotypes [38]. Such constraints on adaptation might be most important in highly variable admixed populations where low-fitness genotypes can be re-created in large numbers each generation. However, it is important to point out that the inferred impotence of selection in this system is not absolute. For example, Fitzpatrick et al. [34,62] show that a few presumably advantageous introduced alleles have become fixed both within the hybrid swarm and in populations far from the Salinas Valley introduction sites. Thus adaptive evolution through gene exchange is happening in this system [63], but so far has done little to improve mean fitness in highly admixed populations.

## Conclusions

Our first- and second- generation hybrid crosses demonstrate that salamander survival was influenced by epistatic genetic interactions, and suggest that non-additive inheritance is an important component of the outcome of hybridization between species in secondary contact. Further, fitness variation among early-generation cross types also indicates that while natural dispersal of a few individuals from the hybrid zone into native populations will not likely result in the rapid displacement of native genotypes, the human-mediated introduction of highly non-native salamanders will dramatically reduce the relative fitness of native salamanders. Lastly, our comparison of early-generation hybrids with contemporary-generation hybrids demonstrates that selection has not been successful in eliminating unfit genotypes from some wild populations, and variation in salamander fitness may be maintained by complex ecological and genetic interactions.

## Methods

### Artificial crosses/Lab rearing

For all line crosses parental stock were selected from our captive-reared breeding colony and consisted of (1) CTS from Great Valley Grasslands State Park (Merced County, CA) and Jepson Prairie Reserve (Solano County, CA), both of which are representatives of the widespread "Central Valley" phylogroup [64]; (2) BTS from the abandoned Five-Star Fish Farm (Lake County, CA) that are known to be derived from the same introductions as the Salinas Valley hybrids and are allopatric from CTS; (3) captive-bred F1 hybrids; and (4) lab-raised wild-collected contemporary hybrids from Pond H (Monterey County, CA). Individuals were selected for breeding crosses based on the presence of secondary sexual characteristics (*e.g*., swollen cloaca and laterally flattened tail for males and distended abdomen for females).

Matings were performed in outdoor aquatic mesocosms (1.8 m-diameter plastic cattle tanks) with 0.6 m^2 ^cotton twine grids to provide substrate for oviposition. Four males of the appropriate genotypic class (e.g., CTS, BTS, or F1) were allowed to acclimate to each mesocosm prior to the presentation of a single female. Mesocosms were subsequently checked daily for the presence of eggs. Upon detection of eggs, the female was transported to a 15 L plastic container in the lab to complete oviposition. Females typically laid eggs for 2-3 days following initial oviposition in the breeding chamber. All eggs were counted and separated into 5.8 L plastic containers with 20% modified Holtfreter's solution [65] until hatching. Egg clutches were monitored daily, and dead/non-developing eggs and hatchlings were removed. We attempted to produce two replicate families per cross-type.

Hatchlings remained in the 5.8 L containers until all surviving eggs had hatched. We haphazardly selected 150 hatchlings from each clutch for the experiment. If a second family was produced we randomly removed 75 of the initially selected individuals and replaced them with the individuals from the second clutch. Hatchlings selected for inclusion were placed in individual 89 ml plastic cups and fed newly hatched Great Salt Lake brine shrimp (*Artemia salina*) *ad libitum*. After a few weeks, surviving larvae were moved to 473 ml plastic cups and transitioned from brine shrimp to California blackworms (*Lumbriculus variegatus*). After a few more weeks, surviving larvae were moved to 5.8 L containers for the duration of the larval period. Due to space limitations and overall high survival, all cross-types were randomly culled to 50 individuals (25 per family if two families were present) per cross type before transitioning to 5.8 L containers. Some large larvae were ultimately moved to 15 L plastic containers until metamorphosis or up to 1 year post-hatching, whichever came first. Each hatchling received a full water change every three days or more frequently as necessary. All individuals were checked daily and dead larvae were removed and preserved in 95% ethanol.

Metamorphosing individuals with reduced gills and caudal fins were placed in 5.8 L containers with moistened sponges to complete metamorphosis. Individuals were monitored daily during metamorphosis for closure of the gill slits and loss of the tail fin, at which time each individual was weighed to the nearest 0.1 gram and assigned a total time from hatching to metamorphosis (Tmet). At the same time we measured total length (TL) and snout-vent length (SVL) to the nearest 1.0 mm. Animals were either euthanized or accessioned into our captive breeding colony, following tissue extraction for future DNA analyses.

### Statistical Analyses

The most important fitness measure is survival. However, among survivors we measured additional predictors of lifetime fitness, including mass and SVL at metamorphosis, Tmet, and growth. Metamorphic mass and SVL are frequently used as predictors of lifetime fitness because they have been shown to affect time to maturity and fecundity [66,67]. Tmet plays an important role in the survival of amphibians with complex life-history patterns because pond drying can be a major source of mortality in seasonal aquatic habitats [68]. Growth combines aspects of size and Tmet and is a key element in amphibian life-history transitions [69,70].

To determine the mode of gene action affecting the expression of phenotypic variation in fitness-related traits, we used line cross analyses, specifically joint scaling tests, as described in Chapter 9 of Lynch and Walsh [71]. Joint-scaling tests use weighted least squares regression to compare observed and expected means and standard errors of each parental species (P1 and P2), with each hybrid cross (backcrosses [B1 & B2], F1, and F2) to parameterize alternative quantitative genetic models of additive, dominance, and epistatic gene action (Table 6). The joint scaling test fits the following multiple regression model to the observed phenotypic family means,(1)

**Table 6:** Parameter coefficients (*i.e*., M matrix; [Lynch and Walsh 1998]) for each of the models.

	Line	*μ*_0_	*α*	*δ*	*α*^2^	*δ*^2^	*αδ*
P1	CTS	1	-1	-1	1	1	1
P2	BTS	1	1	-1	1	1	-1
F1	(P1 × P2) & (P2 × P1)	1	0	1	0	1	0
F2	(P1 × P2) × (P1 × P2)	1	0	0	0	0	0
B1	[P1 × (P1 × P2)] & [(P1 × P2) × P1] = bcCTS	1	-0.5	0	0.25	0	0
B2	[P2 × (P1 × P2)] & [(P1 × P2) × P2] = bcBTS	1	0.5	0	0.25	0	0

The purely additive model (*α*) holds that F1 and F2 trait values should fall at the midpoint of the values for P1 and P2, with values for B1 and B2 crosses falling at the midpoint of the F1 and P1 or P2, respectively.

where the *i*th mean (*z*_*i*_) has coefficient *θ*_*s *_denoting the source index, and coefficient *θ*_*H *_denoting the heterozygosity index [72,73]. The source index contrasts the expected number of P_1 _alleles in a line with the reference population (*i.e*., F2), and the heterozygosity index contrasts the expected number of P_1_P_2 _heterozygotes with the F2 reference population. The regression coefficients represent composite additive (*b*_*S*_), dominance (*b*_*H*_), and epistatic (*b*_*SS*_, *b*_*HH*_, *b*_*SH*_) effects, and the intercept, *μ*_0_, is the mean phenotype of the F2 reference generation. Higher-order interactions or non-genetic components of variation are partitioned into the error term. We sequentially fitted each model, starting with the additive effect only and added dominance and epistatic effects up to the full (saturated) model. When epistasis terms are omitted from the simple models, epistatic variance contributes to the error term. We tested the fit of nested regression models using a goodness-of-fit test statistic [71,74]:(2)

where the degrees of freedom equal the number of families (*k *= 14) minus the number of parameters estimated by the model (up to six for the full model in Eq. 1). Var(*z*_*i*_) is the estimated sampling variance (squared standard error) of the *i*th family mean. We tested genetic models sequentially starting with the simplest additive model. Rejection of the additive model indicates that dominance or epistatic effects are making a significant contribution to phenotypic divergence of the lines. Failure to reject the additive model indicates that differences between loci with additive effects are sufficient to explain the observed divergence. We also tested for differences between each 1^st ^(F1) and 2^nd ^(F2, backcrosses) generation line cross and contemporary (~20^th ^generation) hybrid families using multiple pairwise t-tests with Bonferroni corrections. Mass and SVL were log-transformed (ln [*x *+ 1]), and Tmet and growth were square-root-transformed () to accommodate the statistical assumption of normality.

Individual survival is a binary outcome, so the linear regression approach is not entirely appropriate. Therefore, we also used a generalized linear model with binomial error to fit an analogous model [75]:(3)

As described above for the linear regressions, we performed the joint scaling test of the typical quantitative genetic series starting with the additive effect only and added dominance and epistasis terms up to the full second order polynomial in Eq. 3. For each survival model fit, we used restricted maximum likelihood to account for family membership as a random effect. We also tested for differences in survival among line crosses with pairwise Fisher's exact tests with a sequential Bonferroni adjustment.

To investigate whether contemporary, 20^th ^generation hybrids have greater mean fitness than 1^st ^and 2^nd ^generation hybrids with the same admixture proportions, we compared our results from contemporary hybrid families to weighted averages corresponding to the expected frequencies of each line cross in the initial generations of contact. Our most recent estimate of the admixture proportion in the contemporary Pond H population based on 64 independent genetic markers is 62.1% introduced [34]. Therefore, we used a hypothetical "Generation 0" composed of 62.1% pure introduced BTS and 37.9% pure native CTS. We then calculated the expected frequencies of CTS, F1, and BTS in the first generation of hybridization and the expected frequencies of CTS, backcrosses to CTS (bcCTS), F1, F2, backcrosses to (bcBTS), and BTS in the second generation of hybridization assuming random mating and no selection. These frequencies were then used to calculate weighted averages of mass, SVL, Tmet, growth, and survival as the expected values in those three initial generations. All calculations were performed using R [76].

## Authors' contributions

JRJ participated in the design of the study, collected and analyzed the data, and drafted the manuscript. BMF participated in the conception and design of the study, data analysis, and manuscript preparation. HBS participated in the conception and design of the study and manuscript preparation. All authors read and approved the final manuscript.

## Supplementary Material

Additional file 1 **Line-cross Mass plot**. Observed mean Mass and standard errors for each line cross type plotted against expected values under alternative quantitative genetic models Click here for file

Additional file 2 **Line-cross Snout-Vent Length plot**. Observed mean SVL and standard errors for each line cross type plotted against expected values under alternative quantitative genetic models Click here for file

Additional file 3 **Line-cross Growth plot**. Observed mean Growth and standard errors for each line cross type plotted against expected values under alternative quantitative genetic models Click here for file

Additional file 4 **Line-cross Time-to-Metamorphosis plot**. Observed mean Tmet and standard errors for each line cross type plotted against expected values under alternative quantitative genetic models Click here for file

Additional file 5 **Line-cross photographs**. Images of a typical metamorphosed individual from each source index category Click here for file
