# Supplementary material for: Retention of low-fitness genotypes over six decades of admixture between native and introduced tiger salamanders
Source: BMC Evol Biol. 2010 May 18;10:147. doi: 10.1186/1471-2148-10-147 (PMC2889957; doi:10.1186/1471-2148-10-147)

Additional File 1. Observed mean Mass and standard errors for each line cross type plotted against expected values (solid line) under **A)** additive only model ($\mu_0 + b_S$), **B)** additive + dominance model ($\mu_0 + b_S + b_H$), **C)** additive + dominance + additive epistasis model ($\mu_0 + b_S + b_H + b_{SS}$), or **D)** additive + dominance + additive epistasis + dominance epistasis model ($\mu_0 + b_S + b_H + b_{SS} + b_{HH}$).

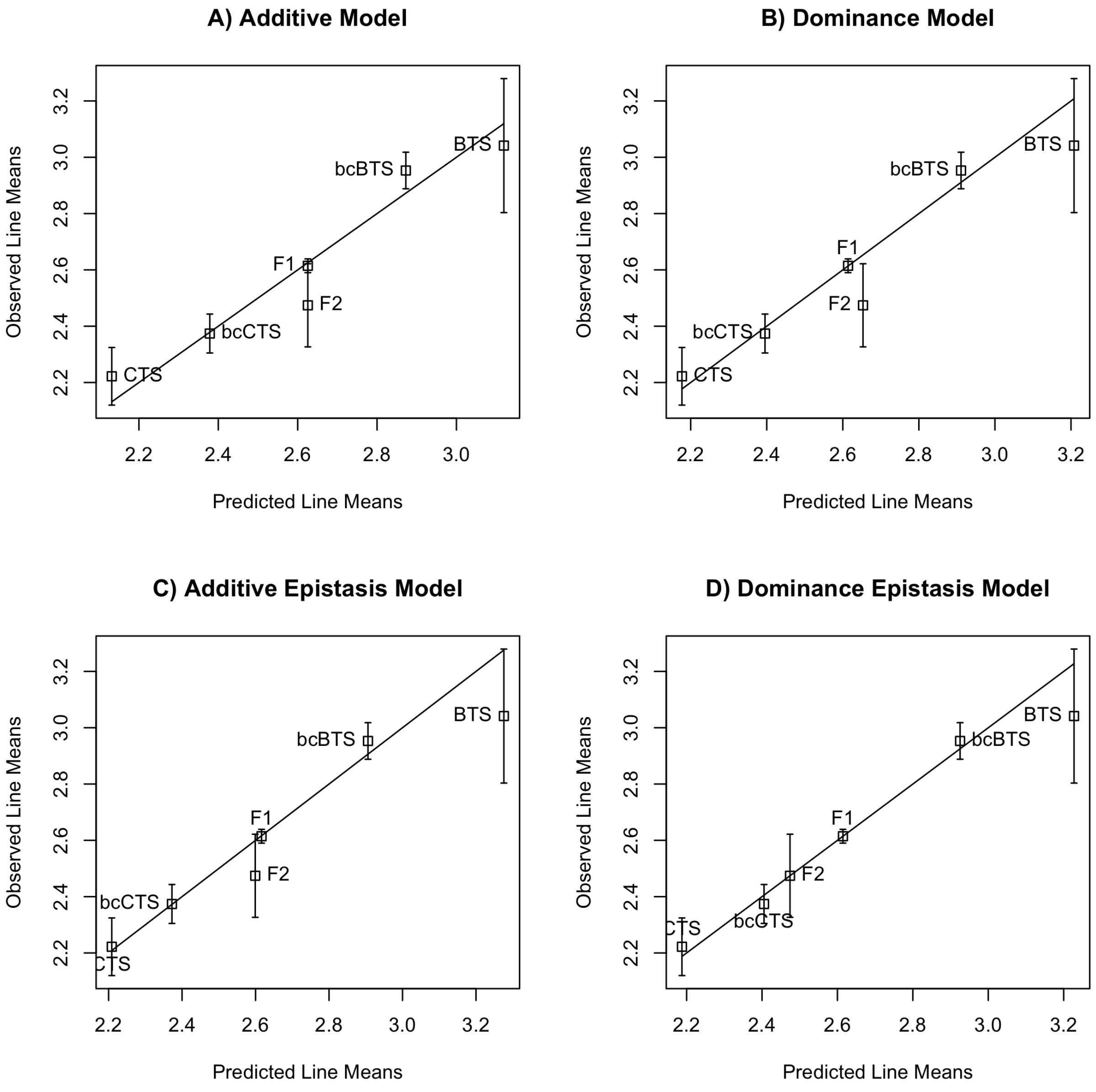

Supplement: Additional file 1 — Line-cross Mass plot. Observed mean Mass and standard errors for each line cross type plotted against expected values under alternative quantitative genetic models [file 1471-2148-10-147-S1.PDF]
