# Supplementary material for: Retention of low-fitness genotypes over six decades of admixture between native and introduced tiger salamanders
Source: BMC Evol Biol. 2010 May 18;10:147. doi: 10.1186/1471-2148-10-147 (PMC2889957; doi:10.1186/1471-2148-10-147)

Additional File 5. Typical metamorphosed individual from each source index category: (A) -1, native California tiger salamander; (B) -0.5, backcross to California tiger salamander; (C) 0, F1; (D) 0.5, backcross to barred tiger salamander ; (E) 1, introduced barred tiger salamander.

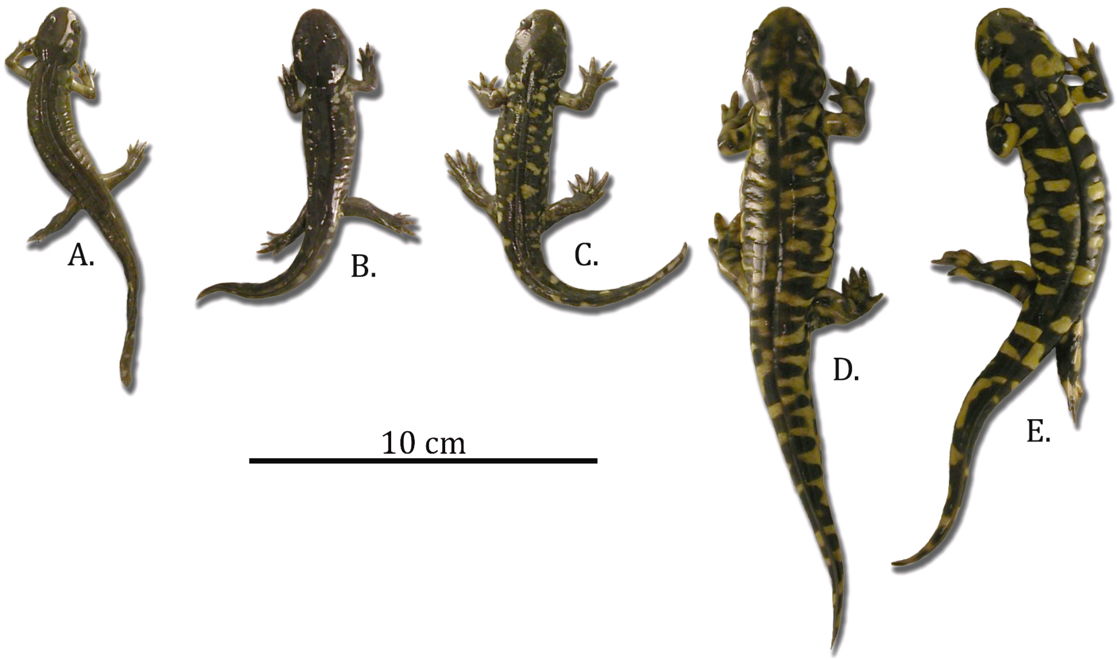

Supplement: Additional file 5 — Line-cross photographs. Images of a typical metamorphosed individual from each source index category [file 1471-2148-10-147-S5.PDF]
